# The HIV-1 Nef Protein Interacts with two components of the 40S small ribosomal subunit, the RPS10 protein and the 18S rRNA

**DOI:** 10.1186/1743-422X-9-103

**Published:** 2012-07-10

**Authors:** Wasim Abbas, Isabelle Dichamp, Georges Herbein

**Affiliations:** 1 Department of Virology, University of Franche-Comte, UPRES EA 4266, IFR 133 INSERM, CHU Besancon, F-25030, Besançon, France

**Keywords:** RPS10, 18S rRNA, tRNA, Nef, HIV, Ribosome

## Abstract

**Background:**

Human immunodeficiency virus type 1 (HIV-1) Nef-encoded protein plays key functions at almost all stages of the viral life cycle, but its role in translation is largely unknown.

**Methods:**

To determine the effect of Nef on translation we used an *in vitro* translation assay. The detection of Nef/RPS10 complexes and the presence of 18S rRNA and tRNAs in the complexes were performed by coimmunoprecipitation and RT-PCR assay.

**Results:**

We observed that the HIV-1 Nef protein specifically impaired translation *in vitro.* We observed the interaction of Nef with RPS10 by coimmunoprecipitation assay. In addition 18S rRNA and tRNAs were present in the Nef/RPS10 complexes.

**Conclusions:**

Our results are consistent with a model in which the Nef protein by binding to two components of the 40S small ribosomal subunit, RPS10 and 18S rRNA, and to a lesser extent to tRNAs, could lead to decreased protein synthesis.

## Background

Ribosome biogenesis involves multiple coordinated steps: synthesis and processing of ribosomal RNA (rRNA), synthesis of ribosomal proteins and their import into the nucleus, the assembly of ribosome subunits, and the transport of the mature 40S and 60S subunits into the cytoplasm [1]. Ribosome biogenesis requires the synthesis, processing and assembly of several protein and RNA components. Structural RNA components of the ribosome include 5S, 5.8S, 18S and 28S rRNA [2]. The 18S, 5.8S and 28S rRNAs are encoded by ribosomal DNA (rDNA) that is organized as expanded chromosome loops in the nucleolus [3]. RNA polymerase I transcribes a single 47S rRNA precursor (pre-rRNA) which is subsequently processed through endonucleolytic and exonucleolytic cleavage into 18S, 5.8S and 28S rRNA [4,5]. Ribosomal proteins are synthesized in the cytoplasm by RNA polymerase II and then imported into the nucleus, where they are assembled into small and large ribosomal subunits [1,2]. The small ribosomal subunit contains one 18S rRNA and approximately 32 ribosomal proteins (known as RPS proteins). The large 60S subunit is composed of one of each 5S, 5.8S and 28S rRNA and approximately 47 ribosomal proteins (known as RPL proteins). The 40S and 60S subunits are then exported into the cytoplasm by exportin-1 and exportin-5 [6], where they assemble with mRNA to form the 80S ribosome. Ribosomes are the organelles that catalyze protein synthesis, and the ribosomal proteins are thought to facilitate the folding and maintenance of an optimal configuration of the rRNAs, favoring ribosome biogenesis and perhaps in this way conferring speed and accuracy for protein synthesis. Extraribosomal functions of ribosomal proteins have recently emerged with the involvement of the ribosomal proteins in cell proliferation, differentiation, apoptosis, cancer and NF-κB-mediated gene expression [7,8].

The small ribosomal protein S10 (RPS10) participates in ribosome biogenesis and is involved in the cellular translational machinery [8,9]. The *RPS10* gene is located on chromosome 6 and contains six exons, with the start codon in exon 2. *RPS10* encodes a 165-amino-acid-long RPS10 protein, a component of the 40S ribosomal subunit [10]. RPS10 protein can be cross-linked to eukaryotic initiation factor 3 (eIF3) of translation, an observation suggesting that RPS10 protein forms part of the domain involved in binding of the initiation factor to the 40S subunit at the start of the translation [11]. Studies of bacteriophage λ transcription identified a NUS (N utilization substance) complex necessary for certain transcription termination events during bacteriophage λ infection. One component of the NUS complex, the host NusE protein, is in fact RPS10 [12]. Recent structural work has shown that RPS10 together with NusB, another host protein, interacts with specific regions of λ transcripts and can do so only when it is not associated with the ribosome [13]. RPS10 has a globular portion that sits at the ribosome surface and an extended loop that penetrates into the small ribosomal subunit. The latter is essential for ribosome function but not for NUS activity. The NUS complex can effect either termination or antitermination depending on the context [14]. Interestingly, the NUS complex functions as an antiterminator for rRNA transcription. Thus, the presence of RPS10 in the NUS complex provides one way in which the rRNA and ribosomal proteins can be coupled, i.e., a deficiency of RPS10 will lead to less antitermination and less rRNA [15,16]. In addition, RPS10 has been recently shown to participate to the maturation of the 18S rRNA in eukaryotic cells by favoring processing of 18S pre-rRNA precursors at cleavage sites A0/1 and A3, leading to the accumulation of 43S pre-rRNA and 18S-E pre-rRNA respectively in case of RPS10 mutation [10,17].

Nef is a 27-kDa HIV-1 protein that is produced early during infection and translated from multiply spliced viral mRNAs [18]. Endogenous Nef may have evolved a number of different, independent functional activities to enhance the replication and survival of the virus within infected cells and to facilitate its spread *in vivo*[19]. Nef enhances virion infectivity and increases viral replication in primary lymphocytes and macrophages [20]. Nef can mediate down-regulation of CD4 cell surface expression, a phenomenon shown to be important for the release of HIV-1 from the cell [20]. Nef can also downregulate the cell surface expression of major histocompatibility complex class I (MHC-I) molecules, an effect found to protect infected cells from killing by cytotoxic T cells [21]. The Nef protein prevents apoptosis of HIV-1-infected T cells [22,23]. Nef expression within macrophages has been reported to favor the recruitment of resting T cells via the secretion of C-C chemokines and to subsequently favor their activation, suggesting a role for Nef in lymphocyte recruitment and activation at sites of viral replication [24].

Identification of new cellular factors that interact with HIV-1 Nef might help elucidate the function of the HIV-1 Nef protein in virus replication and in HIV pathogenesis. We observed the specific inhibition of cellular translation by HIV-1 Nef. Our results indicate that HIV-1 Nef associates with RPS10. We also provide evidence that the Nef/RPS10 complexes contain 18 S rRNA and tRNAs.

## Results

### HIV-1 Nef inhibits translation *in vitro*

**Figure 1:**
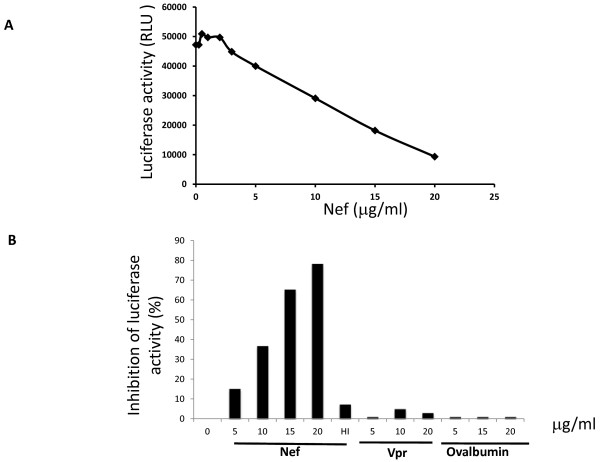
**HIV-1 Nef inhibits translation*in vitro*.** The level of translation of the luciferase mRNA has been measured in the presence of increasing concentrations of rNef, rVpr or recombinant ovalbumin in RRL. Recombinant HIV-1 Vpr and ovalbumin were used to test the specificity of Nef action on translation. In addition an inactive form of Nef was obtained by heating at 100°C for 5 min (HI).

We assessed the effect of Nef on translation efficiency in a rabbit reticulocyte lysate (RRL) system. Increasing amounts of rNef protein were added to a constant amount of RRL. Firefly luciferase encoded by an *in vitro*-synthesized, capped mRNA was used as a translation reporter. After incubation of the RRL with the rNef protein at 4°C, reporter mRNA and amino acids were added, and translation was allowed to proceed by shifting the temperature to 30°C. In pilot experiments, the amount of luciferase protein produced in the RRL, was determined by metabolic labeling (data not shown). Therefore, in all subsequent experiments, the luciferase activity produced in the samples was used as a measure of translation efficiency. Addition of rNef decreased luciferase activity by almost 80% order of magnitude levels of activity observed with control reactions in which amino acids or luciferase mRNA were omitted (Figure 1A and 1B). The inhibition of translation mediated by HIV-1 Nef was dose-dependent (Figure 1A and 1B). Recombinant Vpr and ovalbumin did not inhibit luciferase translation (Figure 1B), indicating a specific effect of HIV-1 Nef on translation.

### HIV-1 Nef interacts with two components of the 40S small ribosomal subunit: the RPS10 protein and the 18S rRNA

**Figure 2:**
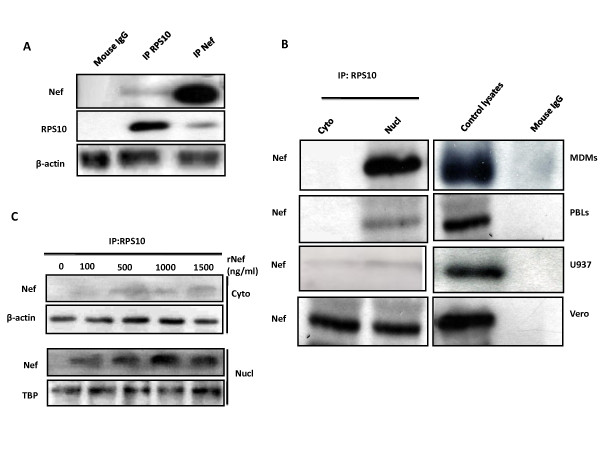
Expression of RPS10/rNef complexes in primary PBMCs, PBLs and MDMs treated with rNef. (**A**) TotalTotal cellular extracts from PBMCs treated with rNef (100 ng/ml) for 2 hours were immunoprecipitated with an anti-RPS10 mAb or an anti-Nef mAb. Immunoprecipitated material was analyzed by western blotting with an anti-Nef mAb or an anti-RPS10 mAb. Results are representative of three independent experiments. (**B**) Cytoplasmic and nuclear extracts from several cell lines (Vero cells, U937 cells), PBLs and MDMs treated with rNef (100 ng/ml) for 2 hours were immunoprecipitated with an anti-RPS10 mAb. Immunoprecipitated material was analyzed by western blotting with an anti-Nef mAb. The controls are input controls from total cell lysates (for IP RPS10 and mouse IgG control). Results are representative of three independent experiments. (**C**) Cytoplasmic and nuclear extracts from U937 cells treated with increasing concentrations of rNef (100–1500 ng/ml) for 2 hours or mock-treated were immunoprecipitated with an anti-RPS10 mAb. Immunoprecipitated material was analyzed by western blotting with an anti-Nef mAb. Results are representative of three independent experiments. β-actin and TBP (TATA binding protein) detection represents input loading controls of the lysates which were used in binding reactions.

Soluble recombinant Nef has been reported to penetrate into cells [25,26]. Following addition of recombinant Nef protein (rNef) to the culture, the endogenous RPS10 protein present in lysates of PBMCs coimmunoprecipitated with rNef (Figure 2A). The amount of coimmunoprecipitated RPS10/rNef complexes detected in total cellular extracts of PBMCs was low, but nevertheless reproducibly noticeable (Figure 2A). Although the interaction between RPS10 and rNef was detected in nuclear and cytoplasmic lysates prepared from Vero cells, most of the RPS10/rNef complexes were present in the nucleus of monocyte-derived macrophages (MDMs), peripheral blood lymphocytes (PBLs), and monocytoid U937 cells (Figure 2B). Input controls from total cell lysates were tested in parallel (Figure 2B). We also observed a mostly nuclear dose-dependent localization of RPS10/rNef complexes in U937 cells treated with rNef (Figure 2C).

**Figure 3:**
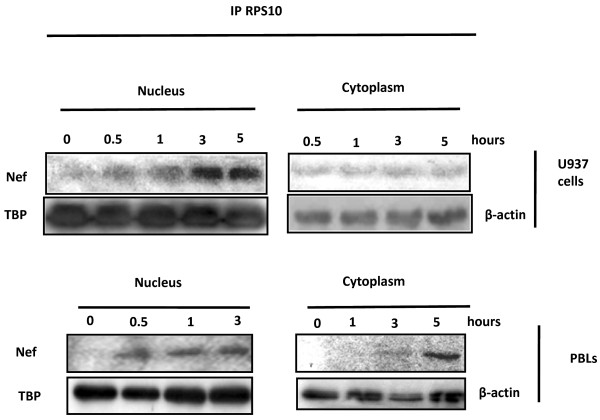
Time course of RPS10/rNef complexes in cells treated with rNef. Cytoplasmic and nuclear extracts from U937 cells and PBLs treated with rNef (100 ng/ml) for up to 5 hours were immunoprecipitated with an anti-RPS10 mAb. Immunoprecipitated material was analyzed by western blotting with an anti-Nef mAb. Results are representative of three independent experiments

Then, we treated U937 cells and PBLs up to 5 hours with rNef, and performed both nuclear and cytoplasmic extracts. Following treatment with rNef of U937 cells and PBLs, a RPS10/rNef complex appears mostly in the nucleus up to 5 hours post-treatment (Figure 3). Our results indicate that in rNef-treated U937 cells or PBLs, a preferential nuclear localization of the RPS10/rNef complexes occurs.

#### Presence of 18s rRNAs and to a lesser extent tRNAs in immunoprecipitated complexes of Nef and RPS10

**Figure 4:**
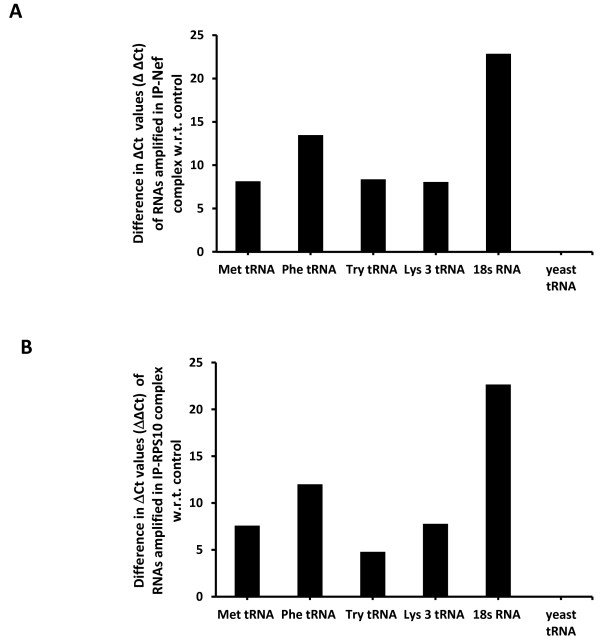
Presence of 18s rRNAs and tRNAs in immunoprecipitated complexes of Nef and RPS10. 18s rRNA and tRNAs are present in Nef (**A**) and RPS10 (**B**) co-immunoprecipitated complexes. MDMs were treated with rNef (100 ng/ml) for 2 h or left untreated (mock). Total cellular extracts were prepared and the detection of tRNAMet, tRNATryp, tRNAPhe, tRNALys3, tRNAyeast (as a negative control) and 18S rRNA was performed in lysates immunoprecipitated with Nef and RPS10 antibodies respectively, followed by RNA extraction and qRT-PCR amplification as previously described [28]. Results are representative of two independent experiments.

Since RPS10 could participate to the processing of the 18 S rRNA maturation [17], we assessed whether 18 S rRNA is part of the RPS10/rNef complex. In addition, the N-terminal Arg-rich region of Nef has been reported to be involved in RNA binding [27]. Thus, experiments were performed to determine if RNAs, especially 18 S rRNA and tRNAs, are present in immunoprecipitated complexes of Nef and RPS10. MDMs were treated with rNef (100 ng/ml) for 2 h or left untreated (mock). Total cellular extracts were prepared and we assessed the presence of tRNAMet, tRNATryp, tRNAPhe, tRNALys3, tRNAyeast and 18 S rRNA in lysates immunoprecipitated with Nef and RPS10 antibodies respectively followed by RNA extraction and qRT-PCR amplification as previously described [28]. We detected the presence of the 18 s rRNA and to a lesser extent of all the tRNAs tested in immunoprecipitated complexes of Nef and RPS10 (Figure 4).

## Discussion

In the present study, we showed that HIV-1 Nef specifically inhibits the translational process. We also observed that the HIV-1 Nef protein binds to two components of the 40S small ribosomal unit, the RPS10 protein and the 18S rRNA, but also to a lesser extent to tRNAs. We observed a preferential nuclear distribution of the RPS10/rNef complexes. Altogether, our results indicate that HIV-1 Nef interferes with the translational process potentially through the interaction with two components of the small ribosomal subunit RPS10 and 18S rRNA.

**Figure 5:**
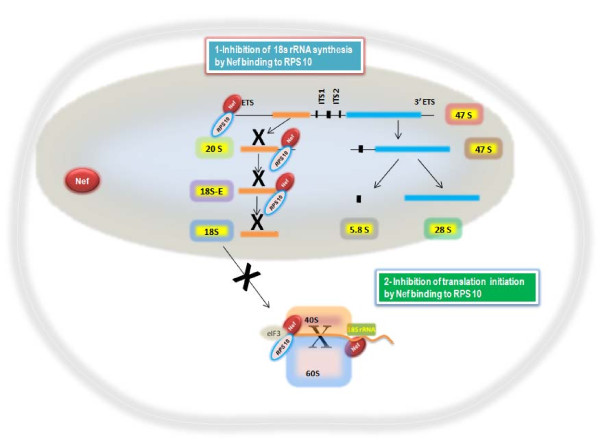
This scheme presents our hypothesis concerning the putative impact of Nef on ribosome biogenesis and on the translational process. The HIV-1 Nef protein binds to RPS10 and to 18S rRNA that could result in at least two distinct features: inhibition of ribosome biogenesis and direct inhibition of the translational process. ETS, external transcribed spacer; ITS, internal transcribed spacers 1 and 2; translation initiation factor eIF3.

Our results indicate that HIV-1 Nef binds to RPS10 and18S rRNA which are components of the 40S small ribosomal subunit. The binding of Nef to RPS10 was concomitant to the accumulation of rNef/RPS10 complexes in the nucleus of cells treated with rNef. In addition, we observed a dose-dependent inhibition of the *in vitro* translation process by HIV-1 rNef, using a RRL assay. Our results suggest that the HIV-1 Nef protein binding to RPS10 and to 18S rRNA could result in at least two distinct features: inhibition of ribosome biogenesis and/or direct inhibition of the translation process (Figure 5).

The RPS10 binding by Nef in the nucleus of the cell could block the nuclear-cytoplasmic shuttling of the 40S small ribosomal subunit including RPS10 and 18S rRNA. Since rNef binds to both RPS10 and 18S rRNA, we hypothesized that 18S rRNA or one of its precursors is the most likely candidate to participate to the Nef/RPS10 interaction. Since the RPS10/Nef complexes are mostly nuclear and contain 18S rRNA, they could thereby prevent the cytoplasmic transport of 18S rRNA, especially if it is uncleaved. Our data suggest that HIV-1 Nef could interfere with the ribosome biogenesis by preventing the nuclear maturation of 18S pre-rRNA in part directed by RPS10. In fact Nef could interfere with RPS10 thereby blocking the processing of 18S pre-rRNA maturation at cleavage sites A0/1 or at site 3. The presence of RPS10 in the NUS complex provides one way in which the rRNA and ribosomal proteins can be coupled, i.e., a deficiency of RPS10 will lead to less antitermination and less rRNA synthesized [15,16]. In agreement with the previous observation, RPS10 has been recently suggested to participate to the maturation of the 18S rRNA in eukaryotic cells [17]. The role of the RPS10 protein in pre-RNA processing has been highlighted by knocking down its expression with siRNAs in HeLa cells [10]. Depletion of RPS10 leads to decreased levels of 18S rRNA, indicating that it is necessary for production of the 40S small subunit. Knockdown of RPS10 expression leads to the accumulation of 18S-E pre-rRNAs, which indicates defects in cleavages at both ends of the 18S rRNA. The mutations of RPS10 with elevated levels of 18E- pre-rRNA has been observed in Diamond-Blackfan anemia, an inherited bone marrow failure syndrome characterized by anemia that usually presents before the first birthday or in early childhood, and is associated with birth defects and increased risk of cancer. The most direct consequence of RPS10 mutation could be a defect in ribosome synthesis. A defect in ribosome biogenesis may also alter regulation and efficacy of translation by affecting the rate of ribosome production and ribosome quality. Thus, the effect of RPS10 on 18S rRNA maturation could be impaired by HIV-1 Nef that will result in a defect in ribosome biogenesis. Therefore, our current view is that a RPS10-18S pre-rRNA complex is recognized by the Nef protein in the nucleus of infected cells which blocks 18S pre-rRNA processing and transfer into the cytoplasm. Future studies will be needed to further test this hypothesis.

HIV-1 Nef modulates cellular signaling by interfering with several pathways, especially the TNF receptor pathway, the CD28 pathway and by modulating the trafficking of the CD4, MHC class I and class II molecules [24]. Nevertheless, we cannot exclude that HIV-1 Nef directly interferes with the translation process. In agreement with this hypothesis, we observed that HIV-1 Nef directly inhibits *in vitro* translation, suggesting a direct effect of Nef on the translational machinery in the cytoplasm of the cell. We detected the presence of tRNAs in the Nef/RPS10 complexes and Nef has been shown to bind tRNAs [29]. In addition, it has been shown that the RPS10 protein can be cross-linked to eukaryotic initiation factor 3 eIF3, an observation suggesting that the RPS10 protein forms part of the domain involved in binding of the initiation factor to the 40S subunit at the start of the translation [11]. Thus, HIV-1 Nef by binding to RPS10 could inhibit the initiation of translation.

## Conclusions

Our results indicate that HIV-1 Nef interacts with two components of the 40S small ribosomal subunit, RPS10 and 18S rRNA. In addition HIV-1 Nef and RPS10 interact with tRNAs, indicating a potential important role for Nef in the control of translation in the infected cells. Future studies will help to delineate more precisely the molecular mechanisms involved.

## Methods

### Peripheral Blood sampling

Buffy coats were obtained from the Blood Transfusion Centre (EFS, Etablissement Français du Sang, France). The study was in accordance with ethical principles as formulated in the World Medical Association Declaration of Helsinki.

### Cell culture

Vero cells and the promonocytic U937 cells were obtained from the American Tissue Cell Culture Collection (ATCC, Manassas, VA). Vero cells and U937 cells were cultivated in RPMI 1640 supplemented with 10% fetal bovine serum. Peripheral blood mononuclear cells (PBMCs), primary monocyte-derived macrophages (MDMs) and peripheral blood lymphocytes (PBLs) were prepared from peripheral blood of healthy donors and were cultured in RPMI medium supplemented with 10% (v/v) pooled AB human serum (Sigma, Munich, Germany), as previously described [30].

### Recombinant Nef treatment

Cells (5x10^6^ cells) were treated with recombinant myristoylated HIV-1 Nef protein (rNef) (SF2 strain) from Jena Bioscience (cat # PR-382). Cell pellets were collected at various periods of time after treatment with rNef, washed extensively and lyzed before western-blot analysis.

### Isolation of nuclear and cytoplasmic extracts

Isolation of nuclear and cytoplasmic extracts was performed as previously described [26]. Cells were washed with wash buffer (10 mM HEPES (pH 7.6), 10 mM KCl, 2 mM MgCl_2_, 1 mM EDTA). Cell pellets were then incubated on ice with cytoplasmic isolation buffer (10 mM HEPES (pH 7.6), 10 mM KCl, 2 mM MgCl_2_, 1 mM EDTA, 0.02% Nonidet P-40). Cytoplasmic extracts were collected by centrifugation, and the nuclear pellets were washed twice in wash buffer, spun, and incubated for 15 min on ice with nuclear isolation buffer (20 mM HEPES (pH 7.6), 420 mM NaCl, 1.5 mM MgCl_2_, 0.2 mM EDTA, 25% glycerol). Supernatants containing nuclear extracts were collected by centrifugation and stored at −80°C. Protease inhibitors (1 mM DTT, 1 mM PMSF, 1 μg/ml aprotinin, 1 μg/ml leupeptin, 1 μg/ml pepstatin) were added to all solutions. Protein concentration in nuclear and cytoplasmic extracts was determined by the Bradford method using a BioPhotometer (Eppendorf).

### Immunoprecipitation

PBMCs, MDMs, PBLs, U937 cells or Vero cells were left untreated or were treated with rNef for different periods of time. Cell lysates were pre-cleared by adding 50 μl of Protein G Plus/Protein A-Agarose (Calbiochem-Novabiochem, Bad Soden, Germany) during 1 h at 4°C. The cleared supernatants were removed, combined with 10 μg/ml anti-RPS10 antibodies (Gene Tex) and incubated overnight at 4°C. Immune complexes were washed in the presence of protease inhibitors and the bound proteins were eluted with sample buffer and run on a 10% SDS-PAGE gels. SDS-PAGE and western blot analysis were performed using an anti-Nef mAb (Upstate Biotechnologies), according to standard procedures [26]. Western blots were developed with the ECL detection kit (Amersham Pharmacia Biotech).

### Detection of the presence of 18s rRNAs and tRNAs in immunoprecipitated complexes of Nef and RPS10

MDMs were treated with rNef (100 ng/ml) for 2 h or left untreated (mock). Total cellular extracts were prepared and the detection of tRNAMet, tRNATryp, tRNAPhe, tRNALys3, tRNAyeast and 18S rRNA was performed in lysates immunoprecipitated with Nef and RPS10 antibodies respectively followed by RNA extraction and qRT-PCR amplification as previously described [28]. The sequence of the primers (Eurogentec, Belgium) was as follows:

tRNA Met forward 5`-CTGGGCCCATAACCCAGAG-`3

tRNA Met reverse 5`-TAGCAGAGGATGGTTTCGAT-`3

tRNA Tryp forward 5`- GGCTCGTTGGTCTAGGGGTA-`3

tRNA Tryp reverse 5`- GATTTGAACCCGGGACCT-`3

tRNA Phe forward 5`- CCTCCTCAAAGCAATACACTGA-`3

tRNA Phe reverse 5`- GGTGATGTGAGCCCGTCTAA-`3

tRNA Lys 3 forward 5`- ATAGCTCAGTCGGTAGAGCATCA-`3

tRNA Lys 3 reverse 5`- ACAGGGACTTGAACCCTGGAC-`3

Yeast tRNA forward 5`-GCTTAGTGGTAAAGCGATAAATTG-`3

Yeast tRNA reverse 5`- TGCCCTTAATGAGAATCGAA-`3

18S rRNA forward 5`- CGGCTACCACATCCAAGGAA-`3

18S rRNA reverse 5`- GCTGGAATTACCGCGGCT-`3

The ddCt values were also determined separately for untreated cells and averaged below 1 (data not shown).

### In vitro translation assay

Increasing amounts of rNef, rVpr (kindly provided by Dr. Bernard P. Roques, U266 INSERM UMR8600 CNRS, Paris France) or ovalbumin (MP Biomedicals, Solon, OH) were first incubated with rabbit reticulocyte lysate (RRL) for 1 h at 4°C according to manufacturer's instructions (Promega). After this incubation, luciferase-encoding mRNA (250 ng) was added to the reaction mixture and translated according to the manufacturer’s instructions (Promega). Capped mRNA was made according to the manufacturer (mMESSAGE-mMACINE, Ambion) by using either pBS-luciferase or pGL-HIV-1 leader-luciferase as a template for T7 *in vitro* transcription. Uncapped mRNA encoding the firefly luciferase gene was purchased from Promega. Luciferase activity was measured according to standard protocols.

### Statistical analysis

Values are the means of independent experiments. Microsoft Excel was used to construct the plots.

## Abbreviations

PBLs: Peripheral blood lymphocytes; PBMC: Peripheral blood mononuclear cells; MDM: Monocyte-derived macrophages; RPS: Small ribosomal protein.

## Competing interests

The authors declare that they have no competing interests.

## Authors’ contributions

GH, WA and ID designed the research, WA and ID performed the experimental work, GH conducted the data analysis. All authors read and approved the final manuscript.
